# Pain neuroscience education with physical activity improves physical and psychological outcomes in older women with chronic low back pain

**DOI:** 10.1038/s41598-025-23951-7

**Published:** 2025-11-07

**Authors:** Teppei Abiko, Shin Murata, Hiroaki Iwase, Koji Nonaka, Kunihiko Anami, Yuki Kikuchi, Katsuyuki Madoba, Kayoko Shiraiwa

**Affiliations:** 1 Department of Physical Therapy, Faculty of Health Sciences, Kyoto Tachibana University, Kyoto, Japan; 2 Department of Physical Therapy, Faculty of Rehabilitation Science, Kobe International University, Kobe, Japan; 3 Department of Rehabilitation, Faculty of Health Sciences, Naragakuen University, Sango, Japan; 4 Department of General Affairs, Nishi Kyoto Hospital, Kyoto, Japan; 5 Faculty of Health Sciences and Medicine, Bond University, Gold Coast, Australia

**Keywords:** Health care, Risk factors

## Abstract

Chronic low back pain (CLBP) significantly impairs quality of life and increases mortality among older adults, making its effective management essential for healthy aging. This quasi-randomized trial compared Pain Neuroscience Education emphasizing physical activity (PNE-PA) with traditional biomechanical treatments (BM) in older women with CLBP over 12 weeks. Community-dwelling women aged 65–90 years with CLBP were assigned to either group based on recruitment timing, using a double-blinded design. PNE-PA aimed to reduce pain-related fear by educating patients about pain neuroscience and encouraging physical activity. Outcomes included physical function, pain intensity, self-reported disability and psychological factors. Analyses used intention-to-treat and bootstrap resampling methods. Between-group differences were assessed using mean differences, 95% confidence intervals (CI), and Cohen’s d. The PNE-PA group (*n* = 24) showed significantly greater improvements than the BM group (*n* = 18) in Chair Stand Test (95% CI: 1.53 to 7.76, d = 0.88), step count (95% CI: 475.97 to 2550.42, d = 0.87), pain catastrophizing (95% CI: -10.64 to -1.95, d = -0.87), and fear-avoidance beliefs (95% CI: -7.40 to -0.14, d = -0.65). These findings suggest that Pain Neuroscience Education emphasizing physical activity was associated with better physical and psychological outcomes among older women with chronic low back pain.

## Introduction

According to the World Population Prospects 2019, by 2050, one in six people globally will be over the age of 65, compared to one in 11 in 2019^1^. This demographic shift poses a considerable challenge for public healthcare due to the progressive decline associated with aging. Chronic pain, affecting 42% of older individuals globally, is prevalent during old age. Chronic low back pain (CLBP) notably impacts quality of life and increases mortality rates among the elderly^2^. Effective management of LBP is crucial to enhance individual well-being and reduce the healthcare burden^3,4^. Thus, addressing LBP in older adults is crucial for preventing frailty, which diminishes physical and cognitive resilience and heightens susceptibility to adverse health outcomes^5^. The relationship between LBP, aging, and frailty underscores the need for comprehensive management strategies to maintain independence and improve quality of life in this demographic.

The International Association for the Study of Pain defines pain as an undesirable sensory and emotional experience associated with actual or potential tissue damage or described in terms of such damage^6^. Historically, chronic low back pain (CLBP) management has often predominantly adhered to a biomedical model, focusing on structural pathology and physical symptoms. This has led to a diverse array of traditional interventions, including pharmacological treatments (e.g., analgesics, muscle relaxants), surgical procedures (e.g., fusion, discectomy), and various physical therapies (e.g., exercise, manual therapy, electrotherapy)^4,7^. While these approaches can provide symptomatic relief and address specific physical impairments, they often demonstrate limited long-term efficacy, particularly in complex, non-specific CLBP cases, as they may not fully account for the multifaceted nature of persistent pain^4,7^. Recognizing these limitations, chronic pain has increasingly been understood as a complex biopsychosocial issue. The biopsychosocial model, which has gained widespread acceptance over the past decade, emphasizes the profound interplay of biological, psychological, and social factors in the experience and perpetuation of pain^8^. This model underscores that factors beyond tissue pathology, such as cognitive and emotional aspects, profoundly influence pain perception and disability^8^. Indeed, maladaptive psychological responses, including kinesiophobia (fear of movement) and catastrophizing (an exaggerated negative orientation to pain), can significantly amplify and perpetuate chronic pain by contributing to central nervous system deconditioning and activity avoidance^9,10^. Therefore, effective management of chronic pain requires an integrated approach that not only addresses physical symptoms but also explicitly targets these crucial psychological and social dimensions through interventions like pain education.

Emerging as a key intervention within this biopsychosocial framework, Pain Neuroscience Education (PNE) effectively manages chronic LBP, with its efficacy confirmed by a randomized controlled trial (RCT)^11^ and systematic reviews^12^. PNE’s biological mechanism targets neurophysiological processes, helping patients reconceptualize pain as a complex brain output influenced by factors like central sensitization, neuroplasticity, and psychosocial elements. This contrasts with traditional biomechanical treatments (BM) for patients with LBP, which primarily address structural dysfunctions under a biomedical model. While BM can restore physical function, they often neglect the neurobiological and psychological components central to chronic pain.

Despite the recognized benefits of PNE and the growing acceptance of the biopsychosocial model in chronic pain management, important research gaps remain, particularly regarding its application to older adults. Several systematic reviews suggest that PNE alone may have limited efficacy in reducing pain intensity directly^12,13^. Its clinical benefits appear more pronounced when combined with exercise therapies, supporting current guidelines that recommend exercise as the primary treatment for chronic low back pain, with PNE improving adherence to physical activity^13,14^. However, the evidence for combined PNE and exercise interventions specifically in elderly populations remains unclear. For example, recent studies exploring this combination in older adults are often limited to feasibility trials^15,16^ or smaller-scale controlled trials, which highlight the need for larger studies to confirm their findings^17^. Furthermore, prior studies have predominantly focused on psychosocial factors, such as pain and kinesiophobia, when assessing outcomes^16]– [17^. The impact on critical physical outcomes for older adults at risk of frailty, such as objective measures of physical function (e.g., balance and gait) and daily physical activity levels, remains insufficiently evaluated. Specifically, there is a significant lack of high-quality research that comprehensively evaluates how reducing psychological barriers through PNE translates into improvements in physical activity and function, which are key determinants of frailty prevention. Such an approach has significant clinical value for promoting healthy aging and reducing the long-term care burden in aging societies. Therefore, dedicated research is warranted to explore the efficacy of combining PNE with physical activity promotion for older adults with chronic low back pain, focusing on these crucial physical outcomes.

The current study aimed to develop and evaluate a modified PNE program tailored for older adults, which incorporated explicit promotion of physical activity alongside standard pain neuroscience education content. Recognizing the unique physiological and psychological needs of the elderly population, this combined approach was designed not only to address maladaptive pain beliefs but also to actively encourage physical activity, which is essential for frailty prevention and functional maintenance.

To examine its effectiveness, we conducted a quasi-randomized controlled trial comparing this PNE-PA intervention with a traditional biomechanical treatment program. We hypothesized that, compared to the BM group, the PNE-PA group would achieve significantly greater improvements in physical function, daily activity levels, and psychological outcomes related to pain catastrophizing and fear-avoidance beliefs.

## Methods

### Study design

This quasi-RCT allocated participants to interventions based on recruitment timing^18^. Specifically, participants were assigned to groups according to predefined time blocks. Each intervention was conducted in the same months in different years. This time-based allocation allowed separation of interventions by period rather than by individual randomization. An independent individual managed participant recruitment across these years, ensuring they were separate from the interventionists and assessors. Moreover, this study was conducted in a double-blind fashion, with both participants and assessors being blinded to the intervention allocation. For assessor blinding, all outcome measurements were performed by an assessor who was not involved in the delivery of either the PNE-PA or BM intervention. This assessor was kept unaware of the participants’ group assignments and the specific hypotheses of the study to ensure objective data collection. The physical therapists delivering the interventions were aware of the specific treatment protocol they were providing (either PNE-PA or BM) but were blinded to the study’s hypotheses and expected outcomes. This study adhered to the CONSORT guidelines^19^ and to the ethical standards of the institutional and national research committee in accordance with the 1964 Helsinki Declaration and its subsequent amendments, and was approved by the Research Ethics Committee of Kyoto Tachibana University (Approval No. 15 − 11, 17/09/2015) and the research began on September 30, 2015. This study was registered and conducted at the University Hospital Medical Information Network (UMIN), a Japanese clinical trial registry that provides information to the World Health Organization, under registration number (ID: 000044436, 04/06/2021).“Participants were informed regarding the nature of the study, after which written informed consent was obtained from all participants prior to participation.

### Participants

Study participants were recruited through informational posters placed in community centers selected to effectively target the local demographic. This study focused exclusively on older women aged 65 to 90 years. This demographic was selected due to the disproportionately higher prevalence and burden of chronic low back pain (CLBP) observed in older women compared to men, especially at advanced ages. Globally, a higher proportion of women by age 80 report experiencing LBP^3^. Furthermore, large-scale Japanese epidemiological studies consistently show higher CLBP prevalence in women in their 60s and 70s than in age-matched men^20^. Given these demographic trends and the potential for sex-specific physiological and psychosocial factors influencing pain and aging, focusing on older women provides crucial targeted evidence for a particularly vulnerable population.

Eligibility was limited to women aged between the ages of 65 and 90 who had been suffering from self-reported LBP for over 3 months. CLBP was defined as low back pain persisting for at least 3 months, without distinction between primary and secondary classifications as defined by ICD-11^21^.The exclusion criteria were severe neurological symptoms (e.g., radiculopathy and spinal stenosis), cardiac disease, respiratory disease, rheumatism, immunodeficiency, cancer pain, cognitive impairment (score < 24 on the Mini-Mental State Examination), and routine pain medication. Furthermore, individuals with other serious musculoskeletal conditions were excluded considering the potential effects of these conditions on the effectiveness of the intervention and clarity of the results. These criteria were generally identical to those in previous studies^17^. Data were collected at the Yamashina Central Elderly Welfare Center, a facility located in Kyoto, Japan, dedicated to providing services and support for the elderly.

### Interventions

All interventions were conducted face-to-face and in groups. The intervention programs consisted of six 60-minute group sessions delivered biweekly over a 12-week period. All participants were instructed to complete group-specific home exercises tailored to their respective treatment philosophies on a daily basis. Compliance with these home exercises was assessed using a daily logbook.

### PNE-PA

The PNE-PA program was carefully designed to satisfy the specific needs of older individuals. Each session typically involved 30 minutes dedicated to interactive pain neuroscience education and 30 minutes focused on promoting physical activity through discussion and practical strategies. The PNE curriculum focused on promoting understanding of the neurophysiology of pain, differentiating between acute and chronic pain mechanisms (e.g., descending inhibition, central sensitization, neuroplasticity), and addressing how various environmental and psychological factors affect nerve sensitivity. Educational materials included simplified diagrams, analogies (e.g., alarm system analogy for pain), and visual aids (e.g., brochure with key concepts). Repetitive teaching methods, including active questioning, and short quizzes, were utilized to ensure comprehension and retention among older adults. In addition, participants were repeatedly advised that they did not need to pay particular attention to posture. Topics specifically addressed common age-related conditions such as chronic inflammation, osteoporosis, and the impact of aging on pain perception and management, providing a contextual understanding of their condition. Participants were educated on the benefits of physical activity beyond ‘strengthening,’ such as its role in calming the nervous system, improving mood, and enhancing overall vitality. Moreover, the program addressed lifestyle-related conditions common among older adults and the role of increased physical activity in managing these conditions. Participants tracked their progress through a daily logbook, noting their step count and positive activities to visualize achievements and encourage physical activity, which was promoted through the use of step counts as a simple and understandable measure for older adults. Participants were encouraged to gradually increase their steps per day based on their own comfort and functional status, without imposing rigid targets. The PNE-PA program was delivered by a licensed physical therapist with 11 years of clinical experience, who had received training in pain education and had conducted similar programs several times in other elderly groups.

### Biomechanical approach (BM)

In the BM intervention, participants engaged in a structured program comprising a 30-minute educational component and a subsequent 30-minute exercise session. The educational segment provided comprehensive instruction on daily activity precautions, the biomechanical mechanisms underpinning LBP, various LBP classifications, and their corresponding management strategies. All participants received standardized guidance on maintaining a neutral lumbar spine during daily activities, which encompassed classical lumbar spine care education emphasizing the avoidance of excessive lumbar flexion and extension. Practical techniques for lumbar spine protection and postural control during routine tasks were also provided. The exercise session focused on individualized practical exercises, specifically targeting the physical aspects of LBP based on whether individuals presented with flexion- or extension-type LBP. For those with flexion-pattern LBP, the intervention included strengthening exercises for the iliopsoas and paraspinal muscles, supplemented by whole-body elongation exercises. Conversely, participants with extension-pattern LBP performed stretching exercises for the iliopsoas and paraspinal muscles, alongside strengthening exercises for the abdominal musculature. Both cohorts were instructed to perform strengthening exercises for 3 sets of 10–15 repetitions and hold stretches for 30 s for 3 repetitions, on a daily basis at home. Exercise intensity was progressed as tolerated, with a strong emphasis on maintaining proper form. Visual handouts illustrating each exercise were disseminated to all participants. To promote adherence, similar to the PNE group, BM participants maintained a daily logbook to record physical activity, including step counts and other daily endeavors. The BM program was administered by a licensed physical therapist possessing 10 years of clinical experience and a Ph.D. in musculoskeletal physical therapy.

### Measurements

All participants were assessed based on their performance across several physical tests and filled out the self-reported demographic information and a number of questionnaires regarding pain. Measurements were conducted at two time points: at baseline and immediately following the 12 -weeks intervention period (post intervention).”

Grip strength was assessed using a hand-held dynamometer, with the highest value of two attempts for each hand being recorded as an indicator of physical function and muscle strength. Long sitting duration was replaced by the “Sit to Reach Test,” which assessed the participants’ flexibility and ability to reach forward while sitting^22^. This test involved participants sitting on the ground and extending their legs forward to reach their toes, with the distance reached being recorded. The One-Leg Stand test was used to measure the duration participants could maintain a one-leg standing position without support^23^. The Chair Stand in 30 s (CS-30) test was provided to quantify the number of full stands a participant could complete from a seated position within 30 s^24^. The Timed Up and Go test (TUG) was used to determine the time taken for a participant to rise from a chair, walk 3 m, return, and sit down^25^. Step count was measured using a pedometer (YAMASA, EX-500) and obtained from the daily log descriptions entered by the participants. The step count was averaged for weeks 1 and 12 of the intervention.

Several standardized questionnaires were employed to assess the various psychological and physical health dimensions of the included participants. Each questionnaire in Japanese was carefully selected based on its reliability and validity in measuring specific constructs related to the study’s objectives.

### Visual analog scale (VAS)

The VAS is a simple yet effective tool for measuring pain intensity^26^. This tool requires participants to mark a point on a 10-cm line, with one end labeled “No pain” and the other “Worst pain imaginable,” that corresponds to their current level of pain. The VAS score is then determined by measuring the distance from the “No pain” end to the participant’s mark, providing a quantifiable measure of pain intensity.

### Roland–Morris disability questionnaire (RDQ)

The RDQ is specifically designed to assess disability due to lower back pain. It consists of 24 statements that describe various daily activities and feelings^27,28^. Participants indicate which statements apply to them that day, with each affirmative response counting as one point. The total score reflects the level of disability, with higher scores indicating greater disability. The RDQ has been widely used in back pain research given its sensitivity to changes in back pain over time.

### Pain catastrophizing scale (PCS)

The PCS assesses an individual’s tendency to focus on and magnify their pain experiences and to feel helpless when dealing with pain^29,30^. It contains 13 items that participants rate on a scale from 0 (not at all) to 4 (all the time), covering thoughts and feelings related to pain. High PCS scores have been associated with worse pain outcomes and have been linked to increased pain intensity and disability.

### Fear-avoidance beliefs questionnaire (FABQ)

The FABQ measures the degree to which people believe that physical activity and work might cause or exacerbate pain^31,32^. It is divided into two subscales: one focusing on physical activity and the other on work-related activities. Participants rate their agreement with each statement on a scale from 0 (completely disagree) to 6 (completely agree). The FABQ helps identify individuals who may avoid physical activity due to fear of pain, potentially contributing to their physical deconditioning and chronic pain.

### Five-item geriatric depression scale (GDS5)

The GDS5 is a short-form version of the original Geriatric Depression Scale designed to screen for depression in older adults^33^. It consists of five questions that determine the participants’ feelings over the past week. The questions address key symptoms of depression, such as satisfaction with life, feelings of emptiness, and hopelessness. Answers are scored on a yes/no basis, with higher scores indicating a greater likelihood of depressive symptoms. This tool is particularly valued for its brevity and ease of use in clinical and research settings.

### Sample size

To determine the required sample size for our study, our sample size calculations were based on the minimum clinically important difference (MCID) for the change in pain scores as measured by the VAS. The MCID was set at 20 points^34^, which reflected a clinically meaningful difference in pain perception among patients. This decision is supported by previous literature indicating that a change of this magnitude is perceived as significant by patients suffering from similar conditions. Given these parameters, we aimed to achieve a power of 80% (β = 0.20) to detect the MCID with a significance level (α) of 0.05. Based on these criteria, our sample size calculation indicated that a minimum of 17 participants per group would be necessary to detect a statistically significant difference between the intervention and control groups at the desired power and significance level. Therefore, our study was designed to include at least 34 participants in total, equally allocated into either the intervention or control group.

### Statistical analysis

At baseline, comparative analyses between the PNE-PA and BM groups were conducted based on the results of normality tests. To ascertain differences between the two groups, independent samples t-tests were performed for normally distributed data, whereas the Mann–Whitney U test was performed for non-normally distributed data.

This quasi-RCT implemented an intention-to-treat (ITT) analysis to evaluate the effect of the intervention on various health-related outcomes^35^. For this ITT analysis, missing data were handled using multiple imputation techniques. This method involves creating multiple plausible complete datasets by imputing missing values based on observed data and a statistical model. Each of these complete datasets is then analyzed independently, and the results are combined to produce a single, more robust estimate of the treatment effect, thereby accounting for the uncertainty due to missing data. In this study, missing values were imputed 5 times. This approach ensures a comprehensive analysis that accounts for potential biases associated with incomplete data. Recognizing the challenges associated with small sample sizes, including the risk of violating the normality assumption and the potential for increased variability, we opted for a bootstrap method with 1000 replications. This approach allowed for the non-parametric estimation of the distribution of our statistics, enhancing the robustness and reliability of our findings by mitigating the influence of outliers and the assumption of normality^36^. The bootstrap method therefore played a crucial role in our analysis, enabling us to draw more confident conclusions about the effectiveness of the intervention.

To compare the effectiveness of the PNE-PA and BM interventions, we calculated the mean differences, 95% confidence intervals (CIs), and Cohen’s d to quantify the effect size. The effect size was calculated using Cohen’s d, where values of < 0.20, 0.20–0.49, 0.50–0.79, and > 0.80 indicate a trivial, small, medium, and large effect size, respectively^37,38^. While no formal correction for multiple comparisons was applied, our analytic strategy, leveraging the robustness of bootstrap methods, prioritized the reporting of effect sizes and their respective 95% confidence intervals. This approach allowed for a comprehensive interpretation of the clinical significance of each outcome, particularly given our small sample size and non-parametric data assumptions. All statistical analyses were performed using Python3.11.8, with the significant level being set at *p* = 0.05.

## Results

**Fig. 1:**
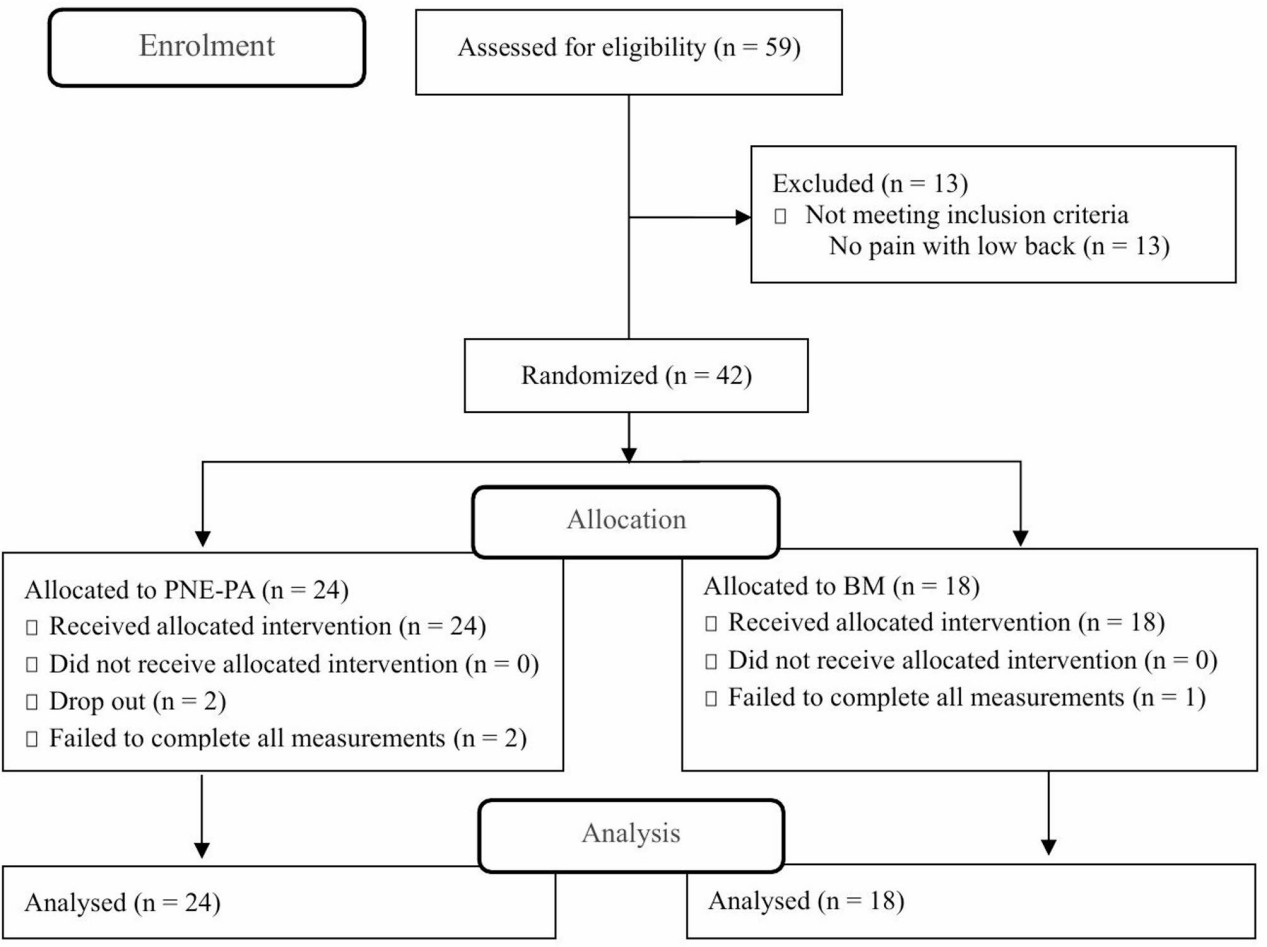
CONSORT flow diagram. PNE, Pain Neuroscientific Education; BM, Biomechanical approach.

Participant flow through the study is depicted in Fig. 1. A total of 59 participants were assessed for eligibility, with 17 excluded due to not meeting inclusion criteria or not having low back pain. Consequently, 42 eligible participants were randomized into two intervention groups: 24 to the PNE-PA group and 18 to the BM group.

Adherence and Attrition: In the PNE-PA group, 2 participants (8.3%) dropped out of the intervention entirely, and an additional 2 participants (8.3%) failed to complete all post-intervention measurements, resulting in a total of 4 participants (16.7%) not having complete outcome data. In the BM group, 1 out of 18 allocated participants (5.6%) failed to complete all measurements. All participants with incomplete data were included in the intention-to-treat analysis, with missing data handled by multiple imputation as described in the Statistical Analysis section. For both groups, daily home exercises were instructed, and compliance was assessed via a daily logbook. While specific quantitative adherence rates were not calculated due to the self-reported nature of the logbook data, all participants who completed the intervention regularly submitted their logbooks. For participants in the BM group, it was confirmed through their daily logbook entries that they performed exercises at least twice a week.

Baseline Characteristics: At baseline, no significant differences were observed across all measured demographic and outcome parameters between the PNE-PA and BM groups, confirming group comparability (Table 1). Intervention Effects: The unadjusted means for all continuous outcomes at baseline and post-intervention are presented in Table 2. Our analysis revealed that the PNE-PA group demonstrated significantly better improvements in physical function compared to the BM group. This was evidenced by a mean increase of 4.53 repetitions in the CS-30 (95% CI: 1.53, 7.76; Cohen’s d = 0.88) and a significant increase of 1476.85 steps in the mean step count (95% CI: 475.97, 2550.42; Cohen’s d = 0.87) (Table 3). Psychological measures also showed significantly better improvements in the PNE-PA group compared to the BM group. This included a significant decrease of 6.37 points in the PCS (95% CI: −10.64, −1.95; Cohen’s d = −0.87) and −3.57 points in the FABQ (95% CI, −7.40, −0.14; Cohen’s d = −0.65) (Table 3). However, PNE-PA did not promote significant changes in pain intensity, as evidenced by a mean increase of 3.25 points (95% CI: −8.98, 15.93; Cohen’s d = 0.14), nor in disability scores with a decrease of − 2.12 points in the RDQ (95% CI: −5.05, 0.27; Cohen’s d = −0.47) and a mean difference of 0.15 point in the GDS5 (95% CI: −0.24, 0.52; Cohen’s d = 0.23). Regarding other outcomes, the PNE-PA group did not show significant improvements compared to the BM group. For physical function measures, the mean differences were: Grip Strength − 0.16 kg (95% CI: −1.66, 1.35; Cohen’s d = −0.10); Sit and Reach 1.09 cm (95% CI: −2.02, 4.09; Cohen’s d = 0.24); One Leg Standing −10.31 s. (95% CI: −26.02, 5.06; Cohen’s d = −0.38); and TUG 0.04 s. (95% CI: −0.21, 0.31; Cohen’s d = 0.11).

**Table 1:** Baseline means for each intervension groups.

	PNE			BM			p-value
	Mean	SD	N	Mean	SD	N	
Age (years)	73.71	6.31	24	70.33	7.08	18	0.11
Height (cm)	153.33	5.71	24	151.44	5.88	18	0.30
Weight (kg)	52.60	10.15	24	47.21	5.85	18	0.06
Grip Strength (kg)	23.92	4.86	23	24.33	3.79	18	0.77
Sit and Reach (cm)	33.98	7.01	24	35.44	11.17	18	0.30
One Leg Standing (sec)	70.13	44.35	24	79.69	36.34	18	0.40
CS-30 (times)	23.21	7.03	24	24.61	5.09	18	0.63
TUG (sec)	5.56	0.67	24	5.50	0.92	18	0.82
Steps	5757.6	2271.5	22	5294.5	2353	18	0.53
Pain Intensity (0–100)	38.26	24.24	23	28.33	26.18	18	0.06
RDQ (0–24)	3.26	4.57	23	2.94	2.86	18	0.69
PCS (0–52)	21.39	12.43	23	22.78	14.13	18	0.74
FABQ (0–30)	15.48	7.17	23	13.89	6.89	18	0.48
GDS5 (0–5)	1.39	0.89	23	1.78	1.00	18	0.09

PNE Pain Neuroscientific Education, BM Biomechanical approach, CS-30 30-seconds chair stand test, TUG Timed Up and Go test, RDQ Roland–Morris Disability Questionnaire, PCS Pain Catastrophizing Scale, FABQ Fear-Avoidance Belief Questionnaire, GDS5 Geriatric depression scale 5.

**Table 2:** Unadjusted means for each intervention groups for all continuous outcomes.

	Baseline						Post intervention					
	PNE			BM			PNE			BM		
	Mean	SD	N	Mean	SD	N	Mean	SD	N	Mean	SD	N
Grip Strength (kg)	23.92	4.86	23	24.33	3.79	18	23.46	5.18	20	24.11	3.47	18
Sit and Reach (cm)	33.98	7.01	24	35.44	11.17	18	33.00	6.25	21	33.53	11.05	17
One Leg Standing (sec)	70.13	44.35	24	79.69	36.34	18	58.67	45.45	21	83.16	42.32	18
CS-30 (times)	23.21	7.03	24	24.61	5.09	18	24.81	5.90	21	21.59	4.89	17
TUG (sec)	5.56	0.67	24	5.50	0.92	18	5.66	0.69	21	5.52	0.99	18
Steps	5757.6	2271.5	22	5294.5	2353	18	6792.40	2525.72	22	4795.89	1933.36	18
Pain Intensity (0–100)	38.26	24.24	23	28.33	26.18	18	29.62	23.90	21	15.56	18.86	18
RDQ (0–24)	3.26	4.57	23	2.94	2.86	18	1.38	1.80	21	3.29	4.51	17
PCS (0–52)	21.39	12.43	23	22.78	14.13	18	14.68	11.39	22	23.88	14.09	17
FABQ (0–30)	15.48	7.17	23	13.89	6.89	18	11.64	6.41	22	13.82	4.56	17
GDS5 (0–5)	1.39	0.89	23	1.78	1.00	18	1.27	0.83	22	1.53	0.87	17

PNE, Pain Neuroscientific Education; BM, Biomechanical approach; CS-30, 30-seconds chair stand test; TUG, Timed Up and Go test; RDQ, Roland–Morris Disability Questionnaire; PCS, Pain Catastrophizing Scale; FABQ, Fear-Avoidance Belief Questionnaire; GDS5, Geriatric depression scale 5.

**Table 3:** Standardized treatment effect sizes with 95% confidence intervals.

	Mean difference	SD	Mean difference			Cohen’s d	
			95% CI			95% CI	
			Low	High	Cohen’s d	Low	High
Grip Strength (kg)	−0.16	1.67	−1.66	1.35	−0.10	−0.69	0.49
Sit and Reach (cm)	1.09	4.60	−2.02	4.09	0.24	−0.40	0.87
One Leg Standing (sec)	−10.31	26.82	−26.02	5.06	−0.38	−1.04	0.25
CS-30 (times)	4.53	5.15	1.53	7.76	0.88	0.38	1.39
TUG (sec)	0.04	0.37	−0.21	0.31	0.11	−0.57	0.77
Steps	1476.85	1687.88	475.97	2550.42	0.87	0.28	1.49
Pain Intensity (0–100)	3.25	23.91	−8.98	15.93	0.14	−0.53	0.72
RDQ (0–24)	−2.12	4.54	−5.05	0.27	−0.47	−0.96	0.11
PCS (0–52)	−6.37	7.29	−10.64	−1.95	−0.87	−1.54	−0.21
FABQ (0–30)	−3.57	5.48	−7.40	−0.14	−0.65	−1.35	−0.02
GDS5 (0–5)	0.15	0.64	−0.24	0.52	0.23	−0.39	0.87

Note: Mean Difference represents the difference in the change from baseline between the two groups (PNE-PA group minus BM group).

PNE, Pain Neuroscientific Education; BM, Biomechanical approach; CS-30, 30-seconds chair stand test; TUG, Timed Up and Go test; RDQ, Roland–Morris Disability Questionnaire; PCS, Pain Catastrophizing Scale; FABQ, Fear-Avoidance Belief Questionnaire; GDS5, Geriatric depression scale 5.

No adverse events or unintended effects were reported in any of the groups throughout the study, indicating that both interventions were well-tolerated by all participants.

## Discussion and conclusions

The present study provides evidence that a PNE-PA intervention, designed to enhance physical activity, resulted in significantly greater improvements in both physical and psychosocial outcomes than a biomechanical intervention among older women with CLBP. Employing a quasi-randomized controlled trial design and statistically rigorous methods, including intention-to-treat and bootstrap analyses, this study addresses limitations often associated with small sample sizes. Baseline comparability between groups was established, thereby supporting the validity of the comparative findings. These methodological strengths enhance the study’s contribution to the field of pain rehabilitation.

This study would make a significant contribution by assessing psychological variables, physical function, and physical activity simultaneously. Prior research on PNE in older adults rarely examined these comprehensive outcomes together. Previous studies have primarily emphasized psychological outcomes, paying less attention to the functional and behavioral domains that are critical for preventing frailty. By capturing changes in pain beliefs (PCS, FABQ), physical function (CS-30), and step count, this study provides a more comprehensive understanding of the intervention’s multidimensional effects. This integrated approach is necessary to validate interventions that target both the cognitive and functional aspects of chronic pain.

The significant reductions in PCS and FABQ scores observed in the PNE-PA group are consistent with findings from studies on both younger^11,12,39–41^ and older adults^9^, suggesting that PNE effectively reduces maladaptive pain beliefs across age groups. By targeting misconceptions about pain, PNE-PA may encourage more adaptive attitudes and coping strategies even in older individuals with less severe CLBP. This outcome aligns with the biopsychosocial model of pain, which recognizes the role of cognitive and emotional processes in shaping pain experiences^6,8^. Psychological factors such as kinesiophobia and pain catastrophizing can exacerbate chronic pain via central nervous system mechanisms^9,10^. Addressing these factors through education, as implemented in PNE-PA, may be essential in improving outcomes for this population.

In contrast, the BM approach primarily emphasizes anatomical factors and may overlook psychosocial contributors to pain. This limitation can inadvertently reinforce unhelpful beliefs and increase patient anxiety, as suggested by Ickmans et al.^41^, who reported that traditional back school education could worsen fear and avoidance behaviors. The limited effectiveness of BM may thus be due to its inability to address central sensitization and related psychological mechanisms involved in CLBP.

The PNE-PA program significantly improved physical function, with large effect sizes for CS-30 (d = 0.88) and step count (d = 0.87). These findings indicate its potential to promote an active lifestyle, which is essential for older adults. The increase in physical activity may reflect reductions in fear-avoidance beliefs and pain catastrophizing, supported by the program’s direct emphasis on movement. As Louw et al.^14,42^ noted, PNE is not limited to knowledge dissemination but also targets behavioral change. Given the well-documented benefits of physical activity for managing pain and preventing frailty^43–45^, the observed increase in step count supports the program’s effectiveness in encouraging activity among older adults with CLBP.

In contrast, no significant improvements were observed in pain intensity or RDQ scores, consistent with previous studies showing limited effects of PNE-based interventions on these outcomes^12,14,40,42,46^. This may be because PNE primarily targets cognitive aspects of pain rather than symptom intensity or functional disability. Furthermore, improvements in ADL may require longer intervention period or the addition of targeted physical or psychological strategies.

Among the physical function measures, only CS-30 showed a significant improvement in the PNE-PA group. In contrast, other tests, including grip strength, TUG, and one-leg standing, demonstrated small effect sizes and wide confidence intervals, suggesting minimal and inconsistent effects. One likely explanation is that these tasks involve minimal lumbar movement and are therefore less sensitive to changes in pain-related fear. The CS-30, on the other hand, requires repeated trunk flexion and extension, which are commonly associated with fear and avoidance behavior in individuals with chronic low back pain. Osumi et al.^47^ reported that kinesiophobia is linked to slower and more guarded trunk movement patterns. Thus, the observed improvement in CS-30 may reflect not only enhanced physical function, but also reduced psychological barriers to movement. These findings indicate that the intervention’s effectiveness may be task-specific, with functional gains most apparent in movements that directly challenge fear-related avoidance patterns.

A major strength of this study is its quasi-randomized controlled design, which enabled a valid comparison between interventions despite real-world constraints. The use of intention-to-treat and bootstrap methods enhanced the reliability of findings, particularly given the small sample size. Focusing on older women with CLBP—a group underrepresented in pain research—this study offers targeted evidence for clinical practice. Moreover, the group-based, community-delivered format provides a practical and potentially cost-effective alternative to individualized care models.

Despite its strengths, this study has several limitations. First, the quasi-randomized design—while practical for a community setting—introduces a risk of unmeasured confounding. Although baseline characteristics were balanced, factors such as subtle variations in pain presentation, pain duration, medication use, and comorbidities may have influenced the observed outcomes. Second, the wide age range (65–90 years) may have introduced physiological variability that influenced outcomes despite predefined inclusion criteria. Third, adherence to home exercises was tracked via self-reported logbooks, which may be prone to reporting bias. Finally, the study’s short duration limited our ability to assess the long-term effectiveness of PNE-PA, which is crucial for evaluating its sustainability in chronic pain management.

The findings of this study have several crucial clinical applications. For clinicians, the significant improvements in physical function (CS-30, step count) and psychological outcomes (PCS, FABQ) observed in the PNE-PA group suggest that incorporating pain neuroscience education, particularly with an emphasis on physical activity, is a valuable approach for managing CLBP in older women. This approach can empower patients by reducing their fear-avoidance beliefs and catastrophizing, thereby facilitating greater engagement in physical activity. The observed increase in step count highlights that PNE-PA can lead to tangible behavioural changes, promoting an active lifestyle, which is crucial for preventing frailty and improving overall health in this demographic. For healthcare systems and policymakers, our results support the utility of group-based, community-delivered interventions for CLBP in older adults, offering a potentially cost-effective and accessible alternative to individualized approaches.

In conclusion, this study demonstrates that a PNE-PA intervention can improve both psychosocial and physical outcomes among older women with CLBP, using a quasi-randomized design and rigorous statistical methods. These findings are consistent with results from younger populations, suggesting that PNE-PA has broad applicability across age groups. The significant gains in physical activity, as evidenced by increased step count and CS30 performance, underscore its potential for promoting active aging. No adverse events were reported, indicating that the intervention is safe and well-tolerated. Despite its limitations, the findings of this study demonstrate that PNE-PA has comprehensive effects, simultaneously improving physical function and daily activity levels as well as psychological function. This integrated impact highlights the intervention’s relevance for both symptom management and functional restoration in older adults. Further research should investigate the long-term benefits and their applicability in broader populations.

## Data Availability

The data will be available upon request for purposes of reproducing the results or replicating the procedure. Interested researchers can request access to the data by contacting the corresponding author. The program codes used during the analysis are not publicly available but can be discussed upon request.
